# The consequences of cultural difference: the international medical graduate journey in New Zealand

**DOI:** 10.5116/ijme.6440.0e37

**Published:** 2023-04-28

**Authors:** Mariska M. Mannes, Davinia J. Thornley, Tim J. Wilkinson

**Affiliations:** 1 School of Social Sciences, Media, Film and Communication, University of Otago Dunedin, New Zealand; 2 Department of Medicine, University of Otago Christchurch, New Zealand

**Keywords:** Cultural values, professional values, cross-cultural transition, International Medical Graduates, New Zealand

## Abstract

**Objectives:**

To identify how differences in cultural and professional values between New Zealand born and trained doctors and International Medical Graduates (IMGs) affect the practice and retention of IMGs in New Zealand.

**Methods:**

A mixed-method approach was used. An anonymous 42-item online questionnaire was used to compare participants' cultural and professional values. Participants were 373 New Zealand doctors, 198 IMG, and 25 doctors born and raised elsewhere but who qualified in New Zealand, a group not identified prospectively. The qualitative component used interviews with 14 IMGs to identify cultural challenges faced and with nine New Zealand doctors to identify the challenges they faced working alongside IMGs. Qualitative data were transcribed and analysed thematically.

**Results:**

There were differences in power distance, with the medically qualified in New Zealand doctors having the highest power distance, followed by the IMGs, suggesting a preference for a hierarchical environment at odds with the New Zealand culture. Interviews found cultural differences in communication styles and hierarchy contributed to professional challenges. The cultural transition was difficult for IMGs as they received minimal support. One-third of IMGs acknowledged their behaviours did not fit well in New Zealand. Complaints about IMGs increased when they reverted to default behaviours regarded negatively by New Zealand colleagues or patients.

**Conclusions:**

IMGs are open to change but face a lack of orientation and cultural education opportunities, hindering integration. Residency programs must recognise this disconnect and incorporate cross-cultural programmes in the curriculum. Such programmes would assist the adaption and retention of IMG doctors.

## Introduction

International Medical Graduates (IMGs) in New Zealand are doctors who obtained their primary medical qualification in a country other than New Zealand.^1^ How an IMG navigates professional and cultural value differences, relative to those of their home countries, will impact their journey to practise effectively in New Zealand.

New Zealand's health system is based on a patient-centred care model^2^ as are the health systems in comparable countries.^3^^, ^^4^ In addition, the New Zealand health system adheres to the New Zealand Public Health and Disability Act^5^ and other consumer protections, such as the New Zealand Health Information Privacy Code 2020.^6^ These additional Codes acknowledge individual patients' rights as being integral to patient-centred care and are underpinned by the general cultural values in society, such as individual rights and equality. Working within this system may be confusing and confronting for IMGs who originate from other countries with non-comparable healthcare systems.

One theoretical framework to understand cultural differences is the cross-cultural value dimensions theory. Cross-cultural value dimensions describe the extent to which cultural groups differ from one another in attributes such as values, beliefs, and behaviours and highlight the impact of these differences.^7^^-^^9^ Cultural values are core beliefs and practices from which individuals operate, guiding their behaviour and determining their worldview. These include the value dimensions of power distance – the acceptance or not of unequal distribution of power,^7^^,^^10^^,^^11^ individualism – the degree of interdependence valued,^7^^,^^12^ and high and low context communication – the amount of context versus verbal code preferred to give meaning to messages.^13^^,^^14^

Like cultural values, professional values are guiding principles that influence an individual's behaviour at work. These values are decided by the administration of the workplace and are influenced by legal obligations. Professional and cultural values are difficult to separate as cultural values influence professional values;^7^^,^^8^ both are learned, influence how people behave and are a code by which we live our lives. For example, in the patient-centred model in New Zealand,^2^^,^^15^ autonomy, honest communication, and partnership between patient and practitioner are encouraged,^16^ as is collaboration between practitioners. This model supports a low power distance, individualist and low context health system comparable to the culture in New Zealand.^7^ In contrast, the paternalistic model prefers beneficence, and the control resides with the practitioner representing high power distance, high context and a collective health system in which family are not separate from the individual.^17^^, ^^18^

Like many OECD countries, New Zealand relies on IMGs^19^^, ^^20^ to fill medical practitioner shortages. For example, of the newly registered doctors in 2019/2020, 1,019 were IMGs compared with 530 New Zealand-trained doctors.^21^ New Zealand accepts IMGs from comparable and incomparable health systems; for 2019/2020, IMGs with current practising certificates comprised 40% of the health workforce, with 28% of IMGs from comparable health systems and 12% from incomparable health systems.^21^ However, the retention rate of IMGs is less than optimal; approximately 60% of IMGs leave New Zealand in the first two years after registering, a trend that has been stable for the last ten years.^22^

The Medical Council of New Zealand (MCNZ) determines which countries have a comparable health system to that of New Zealand and currently lists 23 countries.^23^ Of these, five share English as their primary language: Australia, the United Kingdom, Canada, the United States, and the Republic of Ireland. Despite sharing a common language and having similar underlying societal values to New Zealand and each other, these countries are still culturally diverse.^7^^,^^10^^,^^11^ Doctors from the remaining^18^ countries face differences in language and greater cultural differences.^7^^,^^11^ Doctors from incomparable systems may need to unlearn existing medical skills and learn professional and communication skills appropriate to their new environment. Consequently, their pathway to practise effectively and safely in New Zealand is more involved and is lengthier.^23^^,^^24^ Conversely, IMGs from comparable health systems will require less adjustment.

Much cross-cultural research agrees that misunderstandings occur when people with diverse cultural norms interact,^7^^,^^11^^,^^25^^-^^29^ and findings are similar to research regarding IMGs.^29^^-^^32^ Given this diversity, there is a need to identify and understand how both cultural and professional value differences affect practising and successful integration, especially when relying on IMGs to comprise a considerable portion of the workforce.

Much of the research concerning IMGs focuses on the professional challenges they face transitioning into the Western way of practising.^33^ Less attention is given to how personal cultural values impact the IMGs integration journey to practice successfully.

Therefore, the aim of the study was to identify how cultural and professional values differences create challenges for IMGs practising in New Zealand and for New Zealand doctors working alongside IMGs. Another goal was to gather recommendations for an improved IMG journey, with a view to strengthening retention of IMGs.

## Methods

This study used a theory-informed inductive framework^34^ within an interpretivist paradigm. Theory related to cross-cultural values shaped the research questions and data analysis. Inductive analysis was conducted on data that was not immediately related to the selected cross-cultural value dimension theory to enable unexpected findings to be identified. The study used both quantitative (Phase 1) and qualitative (Phase 2) research methods. Ethics approval was obtained from the University of Otago Human Ethics Committee and the relevant Māori (Ngai Tahu) Research Consultation Committee^35^ for both phases of this research. The primary ethical considerations were the anonymity of all participants and the vulnerability of the participants interviewed.

Two participant groups were prospectively characterised in the study design: (1) doctors born, raised and medically qualified in New Zealand (New Zealand doctors), and (2) doctors who obtained their primary medical qualification in a country other than New Zealand, International Medical Graduates (IMGs). After demographic data were collected, a third group was identified retrospectively, doctors born and raised outside New Zealand but who gained their medical qualification in New Zealand (MQNZ), and their information was included in the data analysis.

### Phase 1 – Professional and Cultural Values Continuum (PCVC) questionnaire

A 42-item anonymous online questionnaire was used to rate the professional and cultural values of New Zealand and IMGs practising in New Zealand. The PCVC questionnaire was designed using a bipolar scale, where opposing characteristics were placed at either end of a seven-point continuum, not unlike Hofstede's^7^ 6-D model. Using bipolar scales minimises any bias toward agreeing with a statement, as both ends of the scale must be considered. To minimise acquiescence and response bias, the polarity of 14 of the 42 items was reversed, and the item order was randomised.^36^

The three cultural value dimensions measured were power distance, individualism, and high and low context communication.^7^^,^^10^^,^^13^^,^^37^^,^^38^ Power distance is the extent to which people within a country expect and accept that power is distributed unequally.^7^^, ^^25^ Individualism reflects the amount of interdependence that is valued.^7^^,^^11^ High context and low context communication measures the amount of context needed to create meaning; this is also referred to as indirect (high) and direct (low) communication.^13^  None of the available cultural values questionnaires was found to be wholly suitable for this study. Selected questions from validated cultural value questionnaires by Singelis and collegaues,^12^ Halverson,^14^ Hofstede,^7^ Gudykunst and colleagues,^38^ Yoo and colleagues,^39^ and Earley & Erez^40^ were used to guide the development of the PCVC cultural values questions in this study, including minor wording changes to better reflect the New Zealand culture and health environment and/or reformat the original question. For each of the three cultural values, ten questions were presented. A participant could score between 10 (low) and 70 (high) points for each value.

The professional values questions needed to be created to accurately reflect the New Zealand healthcare environment. These questions were informed by the Medical Council New Zealand's current Standards of Conduct,^41^ the New Zealand Medical Association Code of Ethics,^42^ and the Code of Professional Conduct for Medical Students at the Universities of Auckland and Otago,^43^ and were underpinned by the New Zealand cultural values. For each of the 12 questions presented, a value of one indicated low commitment, and seven indicated a high commitment to professional values. A participant could score up to 84 points on the professional values continuum. A lower score indicated low commitment and higher scores indicated a high commitment to New Zealand medical professional values.

#### Demographics

Demographic information was also collected and included the following: country of birth; the country in which respondents received their formal education and the country in which respondents qualified as a medical doctor; medical specialisation; the number of years practising; the District Health Board area where the physician was currently practising; ethnicity; and age bracket and gender. Collecting the demographic information allowed for verification that participants were representative of doctors practising in New Zealand. It also meant responses could be grouped to analyse and compare PCVC scores across participant groups.

#### Additional questions

To determine whether cultural differences created challenges for doctors who had not received their formal education nor their medical qualification in New Zealand, these participants were asked two further questions: if English was their first language and the number of years practising in New Zealand. They were also asked to rate the following four questions:

1.    How different is the healthcare system where you came from compared with the New Zealand health care system?

Very different, somewhat different, not very different

2.    How comfortable do your feel working within the New Zealand health care system?

Very comfortable, comfortable, somewhat comfortable, neither comfortable nor uncomfortable, not very comfortable, not at all comfortable

3.    How different have you found the New Zealand culture compared with your own?

Very different, somewhat different, not very different

4.    How much do the cultural differences cause challenges for you in the way you interact in the workplace?

A great deal, a lot, a moderate amount, a little, none at all

To compare how IMGs and MQNZs managed cultural challenges they faced due to cultural differences, we explored their answers to questions three (How different have you found the New Zealand culture compared with your own?) and question four (How much do the cultural differences cause challenges for you in the way you interact in the workplace?). To determine if the responses to those answers differed between the IMG and MQNZ groups, we used a chi-squared test.^44^ Kendall's tau-b test^44^ was applied to assess whether a correlation existed between how these groups answered the two questions.

#### Validation

As well as using questions from existing validated questionnaires (as outlined above), the demographic questions and the additional questions in the PCVC questionnaire were explored for validity in four ways: 1. concordance with the literature review, 2. an interview with an IMG, 3. five peer reviews, and 4. piloting with four New Zealand doctors and five IMGs.

### Phase 2 – Interviews

One-hour, individual semi-structured face-to-face interviews were conducted with IMGs and New Zealand doctors to understand the challenges IMGs faced practising in New Zealand and the challenges New Zealand doctors faced working with IMGs. Participants had completed the PCVC questionnaire from Phase 1 before the interview. Subsequently, it was used to analyse the interview discussion and compare the individual doctor's values with that of the PCVC group results in Phase 1. All interviews were voice-recorded.

Additionally, one-hour interviews were conducted with one Prevocational Educational Supervisor and one Medical Education Coordinator, each from a major District Health Board.

### Study participants and recruitment

Study participants for Phase 1 included New Zealand doctors and IMGs practising in New Zealand in any health setting who had been qualified for two years or more. The MCNZ, Royal New Zealand College of General Practitioners (RNZCGP), and a District Health Board assisted in recruiting doctors.

An electronic information sheet with a link to the questionnaire was provided and the MCNZ agreed to send out the invitation, once only, to a random sample comprising 4000 New Zealand doctors and 1000 IMGs; the RNZCGP posted a paragraph about the study with the link in their online newsletter. Similarly, the District Health Board posted the advertisement on their intranet page. Doctors interested in taking part clicked the link to access more information and the questionnaire. Upon completion of the questionnaire, it was made clear to participants in the survey that clicking Submit implied consent to use their answers for this study. A prize was offered, and to ensure anonymity, participants clicked a separate link to enter the draw after the survey. Additionally, a message inviting participants to be interviewed was shown, and a contact email address provided.

Study participants for Phase 2 included IMGs currently practising in New Zealand for one year or more and who had been qualified for two years plus, and New Zealand doctors practising alongside IMGs. Doctors were recruited from participants who indicated their interest via the Phase 1 questionnaire, through an invitation included in the RNZCGP online newsletter, and via the snowballing method. The researcher approached her connections, and those who agreed were emailed the information sheet and a paragraph about the study to relay to their networks. Any doctor who was interested contacted the researcher directly. Upon agreement to participate, the doctor was sent a link to the PCVC questionnaire with a unique code, the consent form, and a list of broad questions that would guide the interview.

Face-to-face semi-structured interviews were held at a time and place convenient to the participant. While the information sheet indicated to allow one hour for interviews, time was not restricted to allow participants to share as much information as they wished and in their own time. Participants provided written informed consent before the interview commenced and were given the option to turn off the recorder at any time during the interview. Time for a debrief was allowed if the information they shared had caused distress to the participant. The length of the interviews ranged from one hour to one and a half hours.

Data were collected for Phase 1 (questionnaire) from January 2020 to May 2020 and for Phase 2 (interviews) from November 2020 to August 2021.

### Data Analysis

Reversed items from the questionnaire were recoded, and subscores were formed by adding items from each of the four value categories: power distance; individualism; context; and professional values. The mean, standard deviation and 95% confidence intervals were calculated for each subscore across all respondents.

Demographically, most participants were included in the two categories initially defined: 1. doctors raised, educated, and qualified in New Zealand (New Zealand doctors), and 2. doctors born, educated, and qualified overseas, International Medical Graduates (IMGs). However, as mentioned earlier, an unexpected subgroup from the NZ group was identified: doctors who were raised and received their formal education outside of New Zealand but who completed their medical qualifications in New Zealand (MQNZ). Comparisons of mean scores across these three groups was by ANOVA, and correlations between questions was explored using the Chi-square test and Kendall's tau-b test. Significance was set at p < .05.

The qualitative data from the 23 interviews was transcribed verbatim, cleaned to remove any identifiable information, and analysed using theoretical and inductive thematic analysis.^34^ Each transcript was reviewed and analysed, with the investigators identifying responses that informed cross-cultural values theory and identifying themes through patterned responses.^45^ We used the thematic saturation approach by Guest and colleagues^46^  and, using a base size of six interviews, we reached the <5% new information threshold at 8^+2^ interviews. Four further interviews were conducted to ensure diversity of IMGs; no further themes were found. For the theoretical analysis, codes were used to identify terms and comments correlated to the cross-cultural codes: communication, hierarchy and power, culture, difference, and challenges.

## Results

### Phase 1 – Professional and Cultural Values Continuum (PCVC) questionnaire

Of the 659 practising doctors completing the online survey, 596 responses (90%) were complete, usable, and included in the analysis: 62% (n=373) were New Zealand doctors, 4% (n=25) were doctors in the NZ subgroup MQNZ and 34% (n=198) were IMGs. Calculation of the response rate to the initial survey invitation is problematic as the recruitment methods overlapped purely due to the impending COVID-19 pandemic. We have endeavoured to approximate the response rate using dates the complete questionnaires were received as an indicator. For IMGs the estimated response rate was 19% and for New Zealand doctors 10%.

Demographically doctors who completed the survey were practising within all New Zealand's District Health Boards. Furthermore, IMGs were represented in each DHB and came from 31 different countries. The IMGs received their medical qualification in the following countries: Argentina (n=1), Australia (n=6), Bangladesh (n=1), Brazil (n=1), Canada (n=2), China (n=3), Czech Republic (n=1), Demark (n=1), Egypt (n=1), Fiji (n=1), France (n=1), Germany (n=9), India (n=15), Iraq (n=2), Ireland (n=8), Italy (n=1), Netherlands (n=6), Pakistan (n=2), Philippines (n=5), Poland (n=1), Romania (n=1), Russian Federation (n=2), Serbia (n=2), Sierra Leone (n=1), Singapore (n=3), South Africa (n=21), Sri Lanka (n=1), United Kingdom of Great Britain and Northern Ireland (n=183), United States of America (n=15), Uruguay (n=1) and Zimbabwe (n=1). Further demographic data for all doctors is provided in Table 1.

**Table 1.** Demographic Characteristics for Phase 1 Doctors Including New Zealand (n= 373), IMG (n=196), and MQNZ (n=25) doctors Practising in New Zealand in 2021

Variables		New Zealand	IMG	MQNZ	Full sample
Gender					
	Female	201	109	14	324
	Male	172	87	11	270
	Gender Diverse	0	2	0	2
Age Range					
	Below 30	30	13	0	43
	30 - 40	70	43	13	126
	41 - 50	83	70	6	159
	51- 60	107	55	2	164
	61 - 65	51	10	4	65
	66 +	31	8	0	39
Doctor type^1^					
	Specialist	165	91	6	262
	Registrar	45	21	7	73
	Primary Care	4	1	1	6
	Medical Officer	9	3	1	13
	House officer	5	8	0	13
	General Practitioner	139	73	10	222
	Other	6	1	0	7

There were too few IMGs per country to analyse responses by country and therefore all IMG responses were analysed as one group. Table 2 presents the comparison of professional and cultural values in population subgroups according to the PCVC questionnaire.

The results from the Phase 1 study are divided into three parts: 1. cultural values; 2. professional values; and 3. the impact of cultural differences for IMGs.

### Cultural values

As shown in Table 2, despite participant diversity, the questionnaire results did not show statistically significant cultural difference among groups for professional values, individualism, and communication context. However, for power distance, results reveal differences between groups (F_(__2, 596)_ = 3.52, p = .03) with the largest difference being found between the MQNZ and New Zealand group, followed by differences between the MQNZ and IMG groups. For all groups the results indicated a preference for low power distance, with a mean overall score of 24.7 from 70 points.

Generally, all respondents scored close to the midway point along the continuum in the individualism dimension. While MQNZ scored slightly lower, this was not significant. The midway point indicates doctors do not have a strong preference for either individualist or collectivist attributes. There was also no significant difference between groups in the context dimension. All groups scored slightly above the midway point, illustrating that some context was preferred and indicating that they use a slightly more indirect communication style.

### Professional values

As shown in Table 2, overall, study participants scored above the midway point, with an overall mean score of 61.59, and with no significant differences among groups. Although the New Zealand health system is geared towards the individual, the results suggest that most doctors are comfortable involving whānau/family and support members in medical consultations, with all groups scoring just below 3.5/7 for this question. For four questions, all groups scored above six, representing a high commitment to the professional values of the New Zealand health system

Overall results show doctors in this study value lower power distance, use some context in their communication, value both individualistic and collective characteristics, and practise according to professional expectations of the New Zealand health system. However, it must be clear that doctors were categorised into generic groups and looked at as homogenous

### Impact of cultural difference for IMGs

Though doctors practising in New Zealand appear to have similar values to one another, when IMGs were asked in the questionnaire how different they have found the New Zealand culture compared with their own (Q1) and if the cultural differences caused challenges in how they interact in the workplace (Q2), only 40 from 196 IMGs replied, 'none at all'. The MQNZ doctors also were presented with these questions; 14 of the 25 MQNZ doctors answered and therefore, are included in Table 3. Table 3 summarises IMG and MQNZ responses comparing differences between their own culture and the New Zealand culture and whether those cultural differences contributed to challenges faced.

As shown in Table 3, there were no significant differences between IMGs and MQNZ in how they answered Q1 or Q2. For both groups combined, the responses to Q1 were similar to the responses to Q2. For the IMGs, Kendall's tau-b test showed a significant correlation between the answers provided in Q1 with those in Q2 (r_t_ = 0.46, p .001). The MQNZ group was small, so while the correlation was similar this did not reach statistical significance (r _t_ = 0.42, p = .10).

Although the PCVC questionnaire results do not show significant differences between doctors regarding their values, 80% of IMGs agreed that culture caused challenges. The interviews in Phase 2 allow deeper investigation of those challenges.

**Table 2.** Comparison of professional and cultural values in participant subgroups (NZ n=373; IMG n=198; MQNZ n=25) according to the PCVC questionnaire

Value	Group	N	Mean	SD	95% CI	F	p
Professional Values	NZ	373	61.84	6.3	[61.2 - 62.5]	1.36	.258
	IMG	198	61.34	7.01	[60.4 - 62.3]		
	MQNZ	25	59.80	5.42	[57.6 - 62.0]		
	Total	596	61.59	6.52	[61.1 - 62.1]		
Cultural Values							
Power Distance	NZ	373	24.28	5.82	[23.7 - 24.9]	3.52	.030*
	IMG	198	25.30	6.91	[24.3 – 26.2]		
	MQNZ	25	27.08	7.71	[23.9 - 30.3]		
	Total	596	24.73	6.31	[24.2 - 25.2]		
							
Individualism	NZ	373	38	6.96	[37.3 - 38.7]	1.85	.158
	IMG	198	38.14	7.52	[37.1 - 39.2]		
	MQNZ	25	35.28	4.94	[33.2 - 37.3]		
	Total	596	37.93	7.09	[37.4 - 38.5]		
							
High/Low Context	NZ	373	41.89	5.54	[41.3 - 42.5]	1.41	.244
	IMG	198	41.04	6.05	[40.2 - 41.9]		
	MQNZ	25	41.48	7.13	[38.5 - 44.4]		
	Total	596	41.59	5.79	[41.1 - 42.1]		

*Significant at p<.05

[NZ New Zealand; IMG International Medical Graduate; MQNZ Medically Qualified in New Zealand; SD= standard deviation; CI=confidence interval; F=variance; p=probability.]

**Table 3.** Cultural Differences for IMG (n=196) and MQNZ (n=25) Doctors Practising in New Zealand in 2021

Q1 How different have you found the New Zealand culture compared with your own culture?		Q2 How much do the cultural differences cause challenges for you in the way you interact in the workplace?				
IMGs				
	None at all	A little	A moderate amount	A lot	A great deal
Not very	23	23	2	0	0
Somewhat	15	68	19	4	1
Very	2	15	12	8	4
MQNZ				
Not very	2	0	0	0	0
Somewhat	1	2	2	0	1
Very	0	2	2	1	0
Note that two IMGs only answered one of the questions and therefore are omitted					

NZ=New Zealand; MQNZ=Medically Qualified in New Zealand

### Phase 2 Interviews

In total 23 respondents were interviewed, comprising 14 IMGs and nine New Zealand doctors. The New Zealand doctors comprised four registrars, one specialist, three general practitioners and one house officer. The IMGs comprised six specialists, five general practitioners, and three registrars. They came from the United Kingdom, the United States of America, South Africa, South America, and South and Southeast Asia. Thirteen respondents were female, and ten were male. The time practising in New Zealand for the IMGs ranged from one year to over twenty years. On average, the interviews were an hour in duration.

Regardless of the time spent in New Zealand, all IMGs spoke candidly of their journeys and the challenges they had experienced or still are experiencing. Two expressed that they would not remain in New Zealand, and two more that they would likely leave. Most of the IMGs had worked in different countries before arriving in New Zealand; therefore, they already had gained experience in different health systems and cultures. In addition, five IMGs had practised as specialists abroad.

### Challenges faced by IMGs and New Zealand doctors

The challenges faced by IMGs were grouped into four main themes: professional values differences; hierarchy and power; communication; and cultural transition.

#### Professional values differences

Half of the IMGs had qualified in incomparable systems relative to the New Zealand system. However, they had practised in a comparable system before arriving in New Zealand, and therefore had some knowledge of, and may have already adapted somewhat to, the expectations of practising in a patient-centred model as followed in New Zealand. IMGs from incomparable systems discussed the lengthy pathway and bureaucratic difficulties they faced in practising in New Zealand. All IMGs from incomparable systems agreed that their qualification and experience appeared redundant and felt they had to start again. Different ways of interacting with patients was a challenge. For some it required them to practise in contrast to their usual practice. Five IMGs discussed that within the system they came from it was normal to include family in healthcare for consultations, decision-making, and asking for their support regarding the patient. One IMG had a complaint made against them because they shared information with a family member without the patient's permission. These IMGs found it difficult not to be able to call on the patient's family for support.

"I usually relied on family support for clients. So, if I had a problem with clients, I was constantly calling families to come in and try to find strategies to take care of clients. It doesn't work like that here. With some Māori families, it works like that, but with Europeans, it doesn't work like that at all. And I have heard many times - I don't want to know anything about my son or my daughter; it's not my problem, it's the government's problem, and you take care of him. I couldn't believe what I was hearing! If they [client] don't want family involved, we cannot ask families to come to take care of them." (IMG 2 Male)

"In [country] if you suspect there is a serious diagnosis, you don't talk about it, you don't tell the patient, you will tell the patient's relatives about it. … And, if, unfortunately, something serious is being suspected, then the doctor will ask the patient actually to go out and wait in the waiting room and talk to the relative about the diagnosis; no one would break this rule. But if an overseas doctor went to [country] and just started revealing what he is suspecting, he'll be considered as the most unkind doctor. That he didn't even think about how the patient would feel or want the diagnosis." (IMG 4 Male)

A common theme from New Zealand doctors regarding IMGs was the inability to practise in a patient-centred way, especially regarding those who had come from an incomparable system that was more authoritarian and paternalistic. However, it was acknowledged by most New Zealand doctors that it was difficult to articulate what patient-centred meant, as it was so embedded in the New Zealand system.

"…the whole patient centred policies that we have is [sic] so foreign and alien to them [IMGs]. And that's probably one of the most important things they need to learn." (NZ 19 Female)

A common theme among all IMGs is that they were never asked or given the opportunity to share their knowledge or discuss different ways of practising. Many had encountered an ethnocentric attitude from New Zealand colleagues and felt forced to adapt. This attitude created a sense of resentment among IMGs, believing their knowledge and experience held no value in New Zealand.

"It really felt like I had to … earn my spurs and show that I was valid and valuable. Because I think any international trainee is always going to face that, are you as good as one of our guys." (IMG 9 Male)

"The difference here is you are expected to know the culture, you are expected to behave as if you are a kiwi (New Zealander). When you don't, people don't like it." (IMG 3 Female)

The researcher asked all New Zealand doctors interviewed if they had inquired about the system or culture their IMG colleague had come from. The majority said they had not, and those who said they had inquired admitted it was more so out of politeness, not a genuine curiosity to understand the differences or identify the support the IMG might require.

#### Power and Hierarchy

Most IMGs recognised New Zealand as less hierarchical than the countries they practised in. Confidence to practise without referring to a senior colleague was a challenge for some IMGs.

"One positive thing [of hierarchy] is that you know who to speak to as the next in line. And I'm thinking particularly of the junior staff. There's much more of a culture where you asked for help, or you constantly have a conversation between you and the level up. … That was a total culture shock for me when I came here. I came as a postgraduate Y3 working as a house officer here and I was shocked that my colleagues who were PGY1 [postgraduate year 1] were making autonomous decisions and it just not crossing their mind to say should I maybe check this with the next above." (IMG 8 Female)

The low power distance way of interacting with colleagues was recognised by most of the IMGs. Questioning and offering opinions to senior medical colleagues generated mixed feelings about whether this was disrespectful. Those with high power distance revealed they would have difficulty interacting in this manner. The quote below is from a New Zealand doctor who, while not born here, arrived in New Zealand at a very early age, was formally educated and qualified here, but was brought up in a hierarchical cultural family environment with which they identify.

"I think one of the main things that I struggle with on a day-to-day basis, even now, being bold enough to say, 'I'm not sure I agree with that management plan' or 'why don't we do it this way?' Whereas my juniors [NZers] speak out easily, … it's just natural. And they can be completely wrong, but it is respected; the bosses respect that and the team respect that. So, I have been working on that. … I think I'll always have a part of me that needs to be subservient to the hierarchy, and I'm not sure how to break that." (NZ 18 Male)

Commonly agreed by IMGs was the perceived power of their supervisors, as the supervisor had the power to recommend whether IMGs were accepted to practise in New Zealand. Behavioural changes were shared, such as agreeing with everything, not asking for clarifications, not adding to a discussion for fear of saying the wrong thing or not seeking information about the New Zealand system or culture for fear of being perceived as incompetent. A New Zealand doctor who was also a supervisor agrees there is a "power dynamic and that the supervisor will always be seen as a gatekeeper to their progression". (NZ 15 Male)

"But when you're in a position where your entire livelihood going from potentially a locum position or a temporary position, that it is all dependant of you staying in this country … all dependant on that ticking of a box - it absolutely colours your behaviour. I would say that without a shadow of a doubt that I was, for the best part of 18 months, an absolute 'yes man', I would do anything because I knew I needed to get a job." (IMG 9 Male)

#### Communication

Communication challenges was a predominant theme identified by both IMGs and New Zealand doctors. Most IMGs discussed colloquialism and slang, and differences between New Zealand English and that of other English-speaking countries. They saw these more as a curiosity; at times frustrating, but manageable (although, regarding slang, some IMGs admitted to making assumptions about the meaning after listening to the whole message). Nonverbal communication was mentioned briefly by most IMGs as a potential issue, specifically lack of eye contact from Māori and Pacific patients. However, it was the communication high/low context communication style that caused the most difficulties for IMGs. The impact was significant for those IMGs who used a low context, direct communication style not readily accepted in New Zealand. If no adjustment to communication was made, New Zealand doctors perceived those IMGs as rude, blunt, and tactless, resulting in complaints.

"I have been accused of being aggressive, being unhelpful, being an authoritarian, being many things. I have had to reflect on that. Why has no one told me this? I'm 46 - why has no one told me this before?" (IMG 3 Female)

"… some people do need a softer touch such as juniors, so I am mindfully gentle to my juniors, but I am straightforward with my peers. When that happened here, it was taken as a personal attack by my practice manager."  (IMG 1 Female)

The term 'sugar coating' came up often, as did 'beating around the bush' when IMGs discussed the New Zealand communication style. IMGs identified that the New Zealand communication style left them unsure about where they stood with New Zealanders or what they really meant.

"I had to learn how to sugar-coat. … It's impossible for a migrant to know sugar coating. it's like, a new language, you know… so I struggle with the sugar coating. I try to copy, but then I think I can't because I don't know if this is the right occasion to use what somebody has said. It's really annoying." (IMG 3 Female)

"… it's easy to get that surface, and act on it, and miss the deep. Sometimes it's not easy to get what they are meaning because they feel confronted if you ask for more clarification." (IMG 1 Female)

New Zealand doctors acknowledged how difficult it must be for an IMG for whom English was not their first language or who had an accent that was not easily understood. Nevertheless, New Zealanders also believed that their IMG colleagues must have courage to work in healthcare, wherein communication is important.

"… they're actually part of serving a New Zealand which is culturally diverse, so they're often multilingual, and serving people who come from their culture and speak their language, which I'm not able to do." (NZ 21 Female)

"In mental health, there's lots more to say about language itself. And I don't know how people do it, I don't speak any other languages. I think it's very brave to go to another country and learn a new language and do a job that relies so heavily on language." (NZ 16 Female)

#### Cultural transition

Twelve of the 14 IMGs reported a lack of support and orientation, hindering adjustment as they lacked local knowledge of expected norms. Most IMGs admitted it was easier to adapt medically than it was culturally. All had taken part in Māori cultural orientation programmes and, interestingly for some, found connections between their own cultures and Māori culture. However, orientation to the broader medical and social culture in New Zealand was lacking. Most IMGs indicated that if they had better support, for example a cultural mentor, in the first two years, adaptation would be much easier. All IMGs found that social networks took time to develop, and they often felt isolated, with work becoming all-encompassing. The New Zealand doctors could not identify any support networks available for IMGs.  

 "And when I came here, I asked if I was going to have a week of introduction, who was going to show me the way? And they told me, we cannot provide that here… it's almost a year, and now I can understand the dynamics much more than I did. There were many mistakes I have made. Some mistakes were risky for patients. I think people coming from abroad need to be much more supported." (IMG 2 Male)

All IMGs indicated that they expected they would have to adapt and were willing to undergo the processes needed to meet expectations. Before arriving, they had all researched the New Zealand culture, and some had previously practised in or visited New Zealand. Identifying and learning new behaviours and expectations was challenging but was the easier aspect of change; enacting new behaviours each day was the more challenging aspect, and most IMGs experienced this. Some IMGs discussed feeling like an imposter. When stressed or tired, IMGs often reverted to their default behaviours and thus were likely to receive complaints, as these behaviours were regarded negatively by New Zealand colleagues or patients.

"It's exhausting! And then also the problem is that it's a learned behaviour when I'm stressed or exhausted or tired or sleep-deprived or all of the above, I resort back to my normal behaviour. And then that will lead to some difficult conversations. So that's the problem. Are you needing me to change fundamentally who I am, which means that I can't carry on with that façade, and then I am not true to myself." (IMG 5 Female)

The realisation that one's own way of practising and being is not applicable was shared by one-third of the IMGs. It was also commented on by New Zealand doctors when asked about the challenges they thought an IMG might face,

"I didn't know how much it was going to be about starting again, I felt that my background as a [doctor] would be much more important and useful. I found here that I use only a small, tiny part of what I was previously, and I have to build a whole new personality, a whole new way of treating clients, a whole new way to interact with doctors, a whole new way to understand what my role is within the society, everything's different." (IMG 2 Male)

Furthermore, a New Zealand doctor recognised the difficulties an IMG might face.

"Where the goals aren't aligned or the perception not aligned, it takes a lot of skill actually to navigate that, so if your worldview or your framework was wired differently, I imagine that would be very challenging." (NZ 20 Male)

## Discussion

Our study has revealed that IMGs encounter challenges caused by cultural and professional value differences, as do their New Zealand counterparts working alongside them. Indeed, several of the interviewees indicated that they intended to leave, or were thinking of leaving, New Zealand due to the challenges they experienced in adapting. Interestingly, however, the results from the PCVC questionnaire in Phase 1 did not reveal significant value differences between doctors, apart from power distance. This is important, as how power is structured and accepted in culture and workplace significantly influences expected interactions, from how we speak to people to whether we speak out at all.^7^^, ^^47^

It could be argued that the similarities in responses from the PCVC questionnaire among the participant groups may be due to doctors in this study having similar personal values, hence their vocation as a doctor. However, how they enact these values differs from what the interviews captured. This creates a dilemma for IMGs, who may believe they have the professional and cultural values required to practise effectively in New Zealand, only to find that they are performed differently due to cultural differences.^8^^, ^^48^ This also creates a dilemma when identifying the information and support IMGs may need. Understanding how values are interpreted will be important when creating effective orientation programmes to support the integration of IMGs. Additionally, linking IMGs' existing values to New Zealand paradigms may assist learning and transition.

Legal obligations also guide how doctors practise, as found in the individualist-collectivist dimension. IMGs from collectivist cultures and incomparable systems are accustomed to involving family members in the consultation and

decision-making. Conversely, New Zealand's patient-centred practice model is based on individualism, with the individual's rights^6^ being integral in healthcare and, without the expressed permission of the patient, not involving family. Patient-centred care can be defined as "health care that strives to empower patients and their families by providing them with information and education about the patient's health condition and encouraging them to be active participants in the decision-making process"^49^ an individualist characteristic.

Thus, IMGs accustomed to having family support and involvement must learn new strategies in practise and new communication skills as they transition into the New Zealand medical culture. For example, communicating information directly to, and building rapport with, the individual patient rather than the family is essential. Interestingly, all doctors in this study scored around midway on the individual collective continuum. For New Zealand doctors this could reflect the inclusion of Whānau Ora^50^ in the New Zealand health system (Whānau Ora is an approach that places families/whānau at the centre of service delivery, and requires the integration of health, education, and social services to improve outcomes for all families/whānau). The mid-way point suggests that both individualist and collectivist characteristics are valued by all respondents. Recognising this commonality in values, despite differences in interpretation, and applying it to expected practice in New Zealand may assist with the cultural and professional adaptation required for IMGs.

Another shift required is within the dimension of power distance, and responses to this value revealed the greatest difference between groups. IMGs found New Zealand less hierarchical than their country and system of origin, which challenged their usual way of practise. Having practised in more hierarchical environments, IMGs were accustomed to checking with their senior colleagues regarding their decision-making. Adopting a lower power distance working relationship was difficult for these IMGs, and those who continued to check in with their senior colleagues were perceived as lacking competence and confidence. This finding is consistent with other studies.^30^^,^^51^^-^^53^ The inability of an IMG to voice their opinions and conclusions, or the tendency to defer to a senior colleague in healthcare, can have devastating consequences for the person being treated.^52^^,^^54^ This is especially applicable when IMGs are practising in a lower power distance environment in which speaking up is expected. However, the results also indicated there is still hesitancy among many New Zealand doctors to speak up or question a senior physician, and this may be due to the hierarchy found within the culture of healthcare.^55^ Where the difference is significant, training to develop skills in speaking up is vital. IMGs who had the opportunity to discuss practice differences with their supervisor developed a shared understanding and were given support and time to adjust, an important step towards practising effectively.

Communication challenges are highlighted in much research, and our findings are consistent with other New Zealand, Australian, United States, United Kingdom, and Canadian studies.^51^^,^^53^^,^^56^^-^^58^ A specific communication difficulty lies within the dimension of the high (indirect) and low (direct) context communication styles. When doctors use contrasting styles, there is a mismatch in communication and information can be lost or misunderstood, ultimately impacting the patient's safety.^51^^,^^59^ Effective communication with both patients and colleagues in healthcare is paramount.

The New Zealand communication style is somewhat direct and very polite, plotting midway on the high/low context continuum, at odds with the low context style some IMGs used. For these IMGs the New Zealand style caused unease; additionally, they had to learn to look for deeper meaning or ask for clarification to ensure accurate interpretation. However, as mentioned, continuously asking for clarification can be perceived as lacking competence^60^^, ^^61^ and can result in the IMG feeling they lack skills. A further implication is that when the environment is not conducive to repeatedly clarifying, IMGs may ask less and instead guess the answers, leaving them and patients vulnerable.

Furthermore, IMGs who used a low context direct style were perceived negatively by their New Zealand counterparts and would receive more complaints from patients. Again, this raises implications for relationship building and collaboration with colleagues, which is the essence of the patient-centred model. Moreover, being perceived as disrespectful or rude challenges the identity of the IMG. For low-context IMGs, direct communication indicates respect, and having to adapt to a more indirect style may cause the IMGs to feel inauthentic.^62^ Hence, any communication skills training needs to be approached with sensitivity and specificity to identify differences and to provide targeted tools in order to be effective.

Although IMGs from comparable systems encounter fewer challenges than those from incomparable systems, both are expected to adapt. Without cultural orientation, however, IMGs must try to interpret unwritten cultural codes of practice. Having to constantly ask how the system works can be perceived as incompetence, leaving IMGs vulnerable. This is problematic in several ways; for example, IMGs may employ unsafe practice and/or be less effective while trying to understand their working environment. Furthermore, the psychological toll experienced by IMGs can manifest as disempowerment or, as several IMGs indicated, feeling like an imposter and losing confidence. Given that IMGs do not know what they do not know, and at present, their New Zealand peers have difficulty articulating the cultural context of practising in New Zealand's patient-centred model, this problem will likely continue.

A further concern is that if New Zealand medical trainers cannot articulate the unwritten cultural rules and expectations, it will be challenging to identify the training and support IMGs need to settle and resolve any internal conflicts. This might sway their decision on whether to remain.

All the cultural dimensions measured created some challenges. Without addressing these challenges, IMGs are vulnerable. Vulnerability also stemmed from a lack of social support outside of work, leaving IMGs socially isolated. Without a social network, which usually develops over time, work became all-encompassing. A further dilemma in creating social networks for IMGs is that they may need to adjust to a social situation differently from the adjustment made at work. IMGs who were collective struggled more with isolation and making meaningful connections than those who were more individualist. IMGs found that New Zealanders are friendly but harder to connect with on a deeper level. This is consistent with other research.^51^^,^^63^ Some IMGs who practised rurally found it easier to make social connections, especially if the New Zealand doctor introduced them to the community.

This study has highlighted how cultural difference creates challenges and the impact of these challenges; however, it is important to acknowledge limitations. Limitations of this study are that Phase 1 included a small sample. Although the purpose of the Phase 1 questionnaire was to provide a descriptor, a larger sample may have uncovered further data regarding country-specific values. The questionnaire distribution coincided with the onset of COVID-19 in New Zealand, which may have limited sample size.

Further research opportunities include: (1) collecting data from a larger sample of country-specific IMGs to identify differences in cultural and professional values that contribute to their challenges; (2) identifying and understanding the psychological toll that culturally diverse IMGs may experience, and examining how they could overcome internal conflict while adapting to the New Zealand system of practising; and (3) collecting data from a large sample of doctors who were raised and educated in a country other than the one they qualified in, to examine cultural values that contribute to challenges in healthcare practice.

## Conclusions

Results of this study expand the understanding of how professional and cultural differences create challenges for IMGs, while adding the voice of New Zealand doctors working alongside IMGs. In addition, our findings contribute to the limited literature on the experience of IMGs in New Zealand. Cultural and professional differences adversely impacted the efforts of IMGs to settle and practise in New Zealand. Therefore, identifying the cultural and professional values differences between IMGs and New Zealand doctors and population is paramount to developing specific cultural training IMGs need and to incorporating such training in the curriculum. Cross-cultural awareness training for New Zealand doctors is also necessary for this group to understand these differences, the psychological toll IMGs might experience, and how to support their IMG colleagues. Given that several participants intended to leave or were thinking of leaving their positions, implementing improved cultural, professional and personal support systems, such as a cultural mentor, may encourage the retention of IMGs needed to provide healthcare in New Zealand.

### Acknowledgements

The authors wish to thank the participants in this study for generously contributing their time to this research and trusting them with their stories, Associate Professor Hugh Slotten for his support and guidance, and Karen Goa for her editorial assistance.

### Conflict of Interest

The authors declare that they have no conflict of interest.
